# Separate Origins of Ice-Binding Proteins in Antarctic *Chlamydomonas* Species

**DOI:** 10.1371/journal.pone.0059186

**Published:** 2013-03-11

**Authors:** James A. Raymond, Rachael Morgan-Kiss

**Affiliations:** 1 School of Life Sciences, University of Nevada Las Vegas, Las Vegas, Nevada, United States of America; 2 Department of Microbiology, Miami University, Oxford, Ohio, United States of America; University of North Carolina at Charlotte, United States of America

## Abstract

The green alga *Chlamydomonas raudensis* is an important primary producer in a number of ice-covered lakes and ponds in Antarctica. A *C. raudensis* isolate (UWO241) from Lake Bonney in the McMurdo Dry Valleys, like many other Antarctic algae, was found to secrete ice-binding proteins (IBPs), which appear to be essential for survival in icy environments. The IBPs of several Antarctic algae (diatoms, a prymesiophyte, and a prasinophyte) are similar to each other (here designated as type I IBPs) and have been proposed to have bacterial origins. Other IBPs (type II IBPs) that bear no resemblance to type I IBPs, have been found in the Antarctic *Chlamydomonas* sp. CCMP681, a putative snow alga, raising the possibility that chlamydomonad IBPs developed separately from the IBPs of other algae. To test this idea, we obtained the IBP sequences of *C. raudensis* UWO241 by sequencing the transcriptome. A large number of transcripts revealed no sequences resembling type II IBPs. Instead, many isoforms resembling type I IBPs were found, and these most closely matched a hypothetical protein from the bacterium *Stigmatella aurantiaca*. The sequences were confirmed to encode IBPs by the activity of a recombinant protein and by the matching of predicted and observed isoelectric points and molecular weights. Furthermore, a mesophilic sister species, *C. raudensis* SAG49.72, showed no ice-binding activity or PCR products from UWO241 IBP primers. These results confirm that algal IBPs are required for survival in icy habitats and demonstrate that they have diverse origins that are unrelated to the taxonomic positions of the algae. Last, we show that the *C. raudensis* UWO241 IBPs can change the structure of ice in a way that could increase the survivability of cells trapped in the ice.

## Introduction

Ice-binding proteins (IBPs) are secreted by many polar unicellular algae. They appear to be essential for survival because every alga from icy environments examined so far has been found to produce them, while mesophilic algae do not [1]. So far, two major types of algal IBP have been identified. One type (type I) is found in diatoms and many cold-adapted bacteria and fungi [2], and was recently found in two other classes of Antarctic algae, a prasinophyte and a prymnesiophyte [1]. Such proteins or their genes have so far not been found in mesophilic diatoms or other algae, leading to the proposal that polar diatoms and other algae acquired IBP genes by horizontal transfer from bacteria [1]–[3]. The other type of IBP (type II), which is structurally unrelated to the diatom IBPs, has so far been identified only in one chlamydomonad alga, the Antarctic *Chlamydomonas* sp. CCMP681 [4], which appears to be a snow alga [4]. Examination of three other chlamydomonad algae from icy habitats (the Antarctic *Chlamydomonas raudensis* UWO241 (this study), and the snow algae *Chloromonas brevispina* and *Chlamydomonas augustae* (JR, unpublished data)) showed that each had IBP activity. Together, these findings led to the question whether the IBPs of chlamydomonad algae were fundamentally different from those of other algae, possibly with a separate origin. Characterizing the IBPs of other chlamydomonad algae would help to answer this question.

To characterize the IBPs of a second chlamydomonad alga, we selected *C. raudensis* UWO241, a species that was isolated from a permanently ice-covered Antarctic lake [5], [6] and that has been the subject of many studies on the adaptation to extreme conditions, including low light [7], low temperature [8] and high salinity [9]. We obtained the IBP sequences of *C. raudensis* UWO241 by sequencing the transcriptome, confirmed that they encode IBPs, and compared the ice-binding activities of *C. raudensis* UWO241 and a mesophilic sister species. *C. raudensis* UWO241 was found to have many IBP genes, but not the expected type II genes. Our results confirm the association of IBPs with icy habitats and provide further evidence that the IBP genes of polar algae have diverse origins.

## Materials and Methods

### Algal strains

*Chlamydomonas raudensis* strain UWO241 (originally named at the University of Western Ontario [6]) was cultured at Miami University under low temperature/low irradiance conditions and kept in an axenic state by periodic plating of single colonies. It is identical to *C. raudensis* strain CCMP1619 from the National Center for Marine Algae and Microbiota [6], both strains originating from a culture isolated by Neale and Priscu [5]. All cultures were grown in 200 mL glass tubes that were immersed in temperature-regulated aquaria [10]. Unless otherwise mentioned, cells were grown in Bold's Basal Medium (BBM) supplemented with 0.7 M NaCl under ambient CO_2_ and continuous light (60 μmol photons m^−2^ s^−1^) at 8°C.

*Chlamydomonas raudensis* Ettl (SAG49.72) cells were originally isolated from a meadow pool in the Czech Republic [6]. The cells were grown axenically in BBM at 12°C, which is near the low end of its temperature range.

### IBP Sequencing

Many attempts to PCR-amplify IBP genes similar to those in the snow alga *Chlamydomonas* sp. CCMP681 [4] were unsuccessful. We then attempted high throughput sequencing of DNA with a SOLiD 3 system (Applied Biosytems), but essentially all the sequences obtained were mitochondrial sequences. This was attributed to the high AT content of mitochondrial DNA, whose low melting point favors its amplification over that of nuclear DNA. We then turned to high throughput sequencing of cDNA.

To prepare cDNA, cells were grown under the conditions stated above except that the growth temperature was 1°C. Cell pellets were shipped on dry ice to UNLV where RNA was extracted with Trizol (Invitrogen) following the manufacturer's instructions. Normalized cDNA was prepared and sequenced at the Roy J. Carver Biotechnology Center, University of Illinois at Urbana-Champaign. Briefly, messenger RNA was isolated from 50 µg of total RNA with the Oligotex kit (Qiagen, Valencia, CA). The messenger RNA-enriched fraction was converted to a 454 cDNA library and normalized as previously described [11]. The library was quantified using a Qubit fluorometer (Invitrogen) and average fragment sizes were determined by analyzing 1 µl of the samples on a Bioanalyzer (Agilent, CA) using a DNA 7500 chip. The library was diluted to 1×10^6^ molecules/µl. Emulsion-based clonal amplification and sequencing on a 1/4 plate on the 454 Genome Sequencer FLX + system was performed according to the instructions of the manufacturer (454 Life Sciences, Branford, CT). Signal processing and base calling were performed using the bundled 454 Data Analysis Software v2.6.

The sequences were screened for sequences resembling known IBP genes. Those resembling known IBP-like sequences were aligned manually. The sequences of three complete IBP isoforms were confirmed by PCR using DNA as template. The presence of N-terminal signal peptides was predicted with SignalP v. 3.0 [12]. Sequences were aligned with BioEdit [13]. Phylogenetic trees were constructed with Mega 5 [14].

### Production of Recombinant IBP

The amino acid sequence of one of the IBP isoforms (without the signal peptide) was sent to GenScript (Piscataway, NJ) for expression of recombinant protein. The nucleotide sequence was optimized for *E. coli* codons (Fig. S1), modified to encode an N-terminal His-tag, and expressed in *E. coli* using the E3 expression vector. The protein was affinity purified on a Ni column and passed through a 0.22 µm filter, with a final yield of about 4 mg. An electrophoretic gel (Fig. S2A) showed a band of about 24 kDa and a larger band that did not enter the gel, both of which stained positively with anti-HIS antibody (Fig. S2B). The larger band thus appeared to be an aggregate of the monomer. GenScript estimated from the band intensities that the aggregated protein accounted for about 30% of the yield.

### Purification of Native IBP

UWO241 cells were grown at 8°C at Miami University and transported to UNLV, where the culture supernatant was confirmed to have ice-binding activity. An ice-binding fraction was ice-affinity purified by eight cycles of freezing, centrifugation, and thawing, as described previously [15]. The freeze-dried sample, after rehydration, was active. Purity, pI and molecular mass were determined by two-dimensional (2D) electrophoresis at the Nevada Proteomics Center, University of Nevada, Reno, as described previously [2].

### Ice-binding Assays

Ice-binding activity was estimated by observing growth of an ice single crystal (a perfect crystal) submerged in the culture supernatant from cells grown at various temperatures as described previously [16].

The effect of the IBPs on the macrostructure of ice was determined as described previously [1]. The culture medium and the control both consisted of BBM containing 700 mM NaCl, with a final osmolality of 1330 mOsm kg^−1^ (corresponding to a calculated freezing point of −2.42°C). The solutions were cooled to −4.0°C, allowed to freeze slowly for an hour, frozen overnight at −5.9°C, and photographed. Under these conditions, about 41% of the water (2.42/5.9) would be in the liquid state.

## Results

### IBP Activity

**Figure 1:**
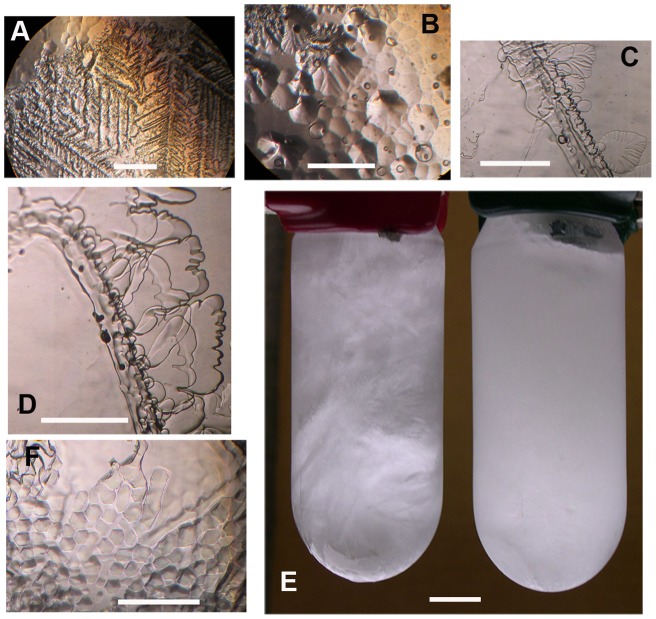
Effects of *Chlamydomonas raudensis* UWO241 ice-binding proteins on ice. A, Irregular ice dendrites growing from a prism face of ice in spent UWO241 medium. B, Pitted basal plane of ice grown in spent UWO241 medium. C, D, Featureless ice crystals (left) grown in the presence of unspent medium (C) and spent SAG49.72 medium (D). E, Effect of UWO241 IBP on the structure of saline ice. Unspent culture medium (700 mM NaCl with 1× Bold) (left) and cell-free spent culture medium containing a natural concentration of UWO241 IBP (right) frozen at −5.9°C. The percentages of liquid and ice in the two tubes are the same (41% and 59%, respectively). Distortion of growing ice by the IBP greatly reduces the size of brine pockets. F, Basal plane of an ice crystal growing in an aqueous solution of recombinant UWO241 isoform 1 IBP. Scale bars, 1 mm, except in E where scale bar is 1 cm.

The cell-free supernatant of a *C. raudensis* UWO241 culture grown at 8°C showed strong ice-binding activity in the form of both irregular dendrites growing from the prism faces and pitting on the ice basal plane (Fig. 1A, B), whereas unspent medium showed no such features (Fig. 1C). IBP was ice-affinity purified from the culture medium and found to be nearly pure (Fig. S3), as well as ice-active, confirming that the source of activity is indeed an ice-binding molecule. In contrast, cell-free supernatant from a mesophilic sister species, *C. raudensis* SAG49.72, grown at 12°C (close to the lower end of its temperature range), showed no activity (Fig. 1D).

The fine structure imposed on growing ice by *C. raudensis* UWO241 IBP can also be seen on a macroscale. Partially frozen culture medium containing a natural concentration of IBP was opaque and had a fine structure, while frozen unspent medium was semi-transparent and had a coarse structure (Fig. 1E), indicating that UWO241 caused the ice to form smaller brine pockets, like those shown between the ice dendrites in Fig. 1A. Such brine pockets may help to prevent drainage of brine from ice, thus preserving a liquid environment for the cells.

### Identification of IBPs

A total of 380,000 cDNA sequences were obtained with an average length of about 400 bp. TBLASTN searches of these sequences found no matches to the type II IBPs of *Chlamydomonas* sp. CCMP681, in agreement with earlier failures to find CCMP681-like sequences by PCR (see Methods). Instead, dozens of sequences resembling type I IBPs were found. These were assembled into about 12 isoforms, 3 of which (GenBank acc. nos. KC012985-KC012987) were complete (Fig. S4). Not all of the incomplete isoforms are necessarily parts of genuine IBPs, as they may be parts of IBP domains in proteins with other functions. Like other IBP genes, each of the complete isoforms has a ∼22-amino acid N-terminal signal peptide, suggesting that they function extracellularly. The calculated sizes and pIs of isoforms 1 and 2 after loss of the signal peptide were about 23 kDa and 4.5, respectively, which agree well with the lower spot on a 2D gel of purified IBP (Fig. S3). The gel has another spot of about twice the size at the same pI, possibly representing a dimer. Isoform 3 has a ∼120 amino acid insert in the N-terminal region, giving it a higher mass (36.3 kDa without the signal peptide). No similar genes are found in the genome of the mesophilic model alga, *Chlamydomonas reinhardtii*.

A recombinant IBP was made from isoform 1 (less signal peptide). The product appeared as a single band of approximately 24 kDa (the expected size) on an electrophoretic gel, although part of the product appeared to aggregate and not enter the gel. A solution of 318 µg ml^−1^ in buffer (50 mM Tris, 150 mM NaCl, pH 8.0) showed clear ice-binding activity (Fig. 1F), confirming that isoform 1 is an IBP.

In a comparison of the DNAs of the two sister species of *C. raudensis*, four primer pairs that amplified sequences containing isoforms 1 and 2 in *C. raudensis* UWO241 either did not amplify similar bands or amplified weak bands of different sizes in *C. raudensis* SAG49.72 (Fig. S5), indicating that the latter is lacking at least some of the IBP isoforms.

**Figure 2:**
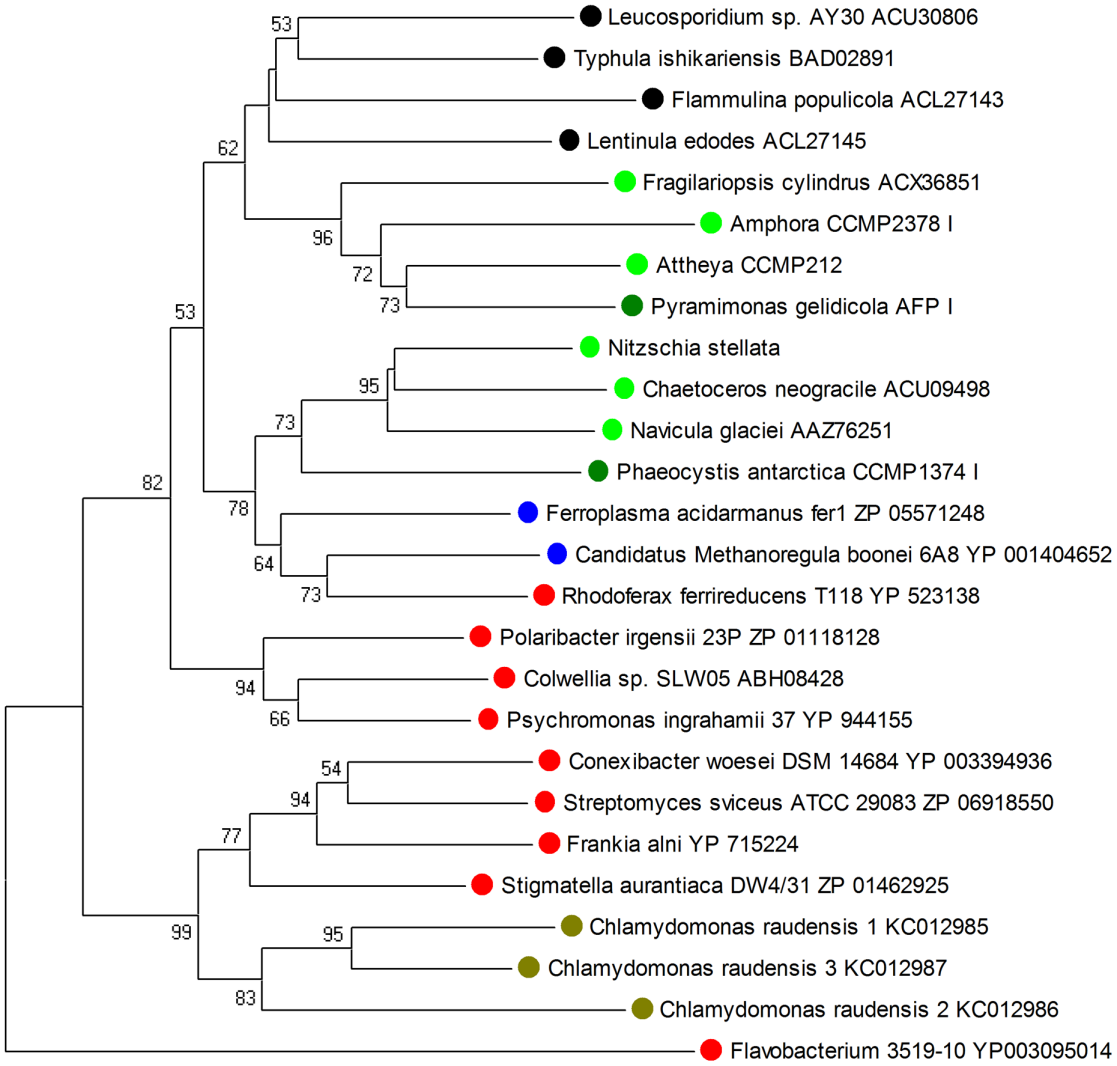
Neighbor-joining tree constructed from amino acid sequences of selected IBP and IBP-like proteins. The *C. raudensis* IBPs (olive) are closest to IBP-like proteins in several bacteria and relatively distant from other algal IBPs. All of the fungal and algal proteins have confirmed ice-binding activities. Among the archaeal and bacterial IBPs, only those of *Colwellia* and *Flavobacterium* 3519-10 have confirmed ice-binding activities. The tree was rooted with the *Flavobacterium* 3519-10 IBP. Numbers at nodes indicate bootstrap values for 500 replications. Values less than 50 are not shown. Colors: black, fungi; light green, diatoms; dark green prasinophyte and prymnesiophyte; blue, archaea; red, bacteria; olive, *C. raudensis*.

In a phylogenetic tree, the UWO241 IBPs clustered with bacterial IBP-like proteins, those from *Stigmatella* and *Frankia* being the closest, rather than with other algal and fungal IBPs (Fig. 2). The four closest matching bacterial proteins in the tree each have other domains of unknown function in addition to an IBP domain, making their functions unclear. However, each has an N-terminal signal peptide, suggesting that they are secreted proteins.

## Discussion

### Function of IBPs

Curiously, *C. raudensis* UWO241 does not encounter ice in its main habitat in Lake Bonney, i.e., the deep euphotic zone, at a depth of about 17 m, where temperatures are around 3–6°C [5]. However, *C. raudensis* (previously called *C. subcaudata*) is widely distributed in Antarctic lakes and ponds [17] and must encounter ice and freezing conditions during its transport between lakes. In fact, *C. raudensis* DNA was recovered from a mixed culture grown from an exposed cyanobacterial mat embedded in the Lake Bonney ice cover, which suggests that viable cells are present in the frozen mats (N. Ketchum & RMK, unpublished data). The mats presumably work their way to the surface by surface ablation [18], where they could be dispersed by winds. In summer, the ice melts at the edges of the lakes, allowing a path for wind-blown cells to enter new bodies of water.

*C. raudensis* SAG49.72 is a mesophilic alga isolated from a meadow pool in the Czech Republic [6]. This species was judged to be identical to *C. raudensis* UWO241 based on cell morphology and internal transcribed spacer (ITS1 and ITS2) and 5.8S rDNA sequences [6]. However, its complete absence of IBP activity (Fig. 1D) and apparent absence of IBP genes (Fig. S5) indicate that the two sister species differ genetically in at least one respect, as well as demonstrate the necessity of IBP genes for survival in icy environments.

The IBPs of *C. raudensis* UWO241 and other polar algae probably serve to increase freezing tolerance, possibly through the preservation of a liquid environment as water freezes. During the summer, liquid water inclusions can make up 40% of the ice cover volume of Dry Valley lakes, which form nutrient-rich habitats for diverse microbial consortia [19]. The small brine pockets that form in the presence of IBPs (Fig. 1E), a consequence of the highly distorted growth imposed on ice by the IBPs (Fig. 1A), are more resistant to draining [4]. A similar phenomenon has been observed in the Arctic, where extracellular polymeric substances, possibly a glycoprotein, excreted by the sea ice diatom *Melosira arctica* created convoluted ice-pore morphologies in sea ice, potentially increasing its habitability and primary productivity [20]. The IBPs of other ice-associated algae, *Chlamydomonas* sp. CCMP681 and *Phaeocystis antarctica* have similar effects on the structure of sea ice [1], [4]. *P. antarctica*, when frozen in sea ice, has an unusually high freeze-thaw tolerance [21], possibly because of this fine structure.

### Origins of IBPs

In the case of polar algae with type I IBPs (presently including diatoms, a prasinophyte and a prymnesiophyte), a number of observations suggest a polyphyletic origin of their IBP genes, with the donors likely being bacteria [1]–[3]. These include high similarities between the algal and bacterial genes, an apparent absence of similar genes in closely related species from warmer regions, a strong incongruence between phylogenetic trees based on IBP and 18S rRNA sequences, and an absence of introns. (The frequency of introns in the *C. raudensis* IBPs is unclear). By contrast, the type II IBPs of *Chlamydomonas* sp. CCMP681 have numerous introns. The differences in sequence and gene structure suggested that chlamydomonad IBPs might have a different origin. However, the present findings show that this is not the case, i.e., that the *C. raudensis* UWO241 IBPs are type I IBPs that are most closely related to bacterial proteins that cluster separately from other bacterial IBP genes (Fig. 2). The closest match was between isoform 3 and a type I IBP-like protein from *Stigmatella aurantiaca* (ZP-0146295) with 54% identity and 67% similarity over a 214-amino acid region (Fig. S4). As in the previous study [1], the positions of the algal species in the IBP tree (Fig. 2) bear almost no resemblance to the their positions in an 18S rRNA tree (Fig. S6), providing further evidence against a monophyletic origin of the *C. raudensis* IBP genes. These results suggest, but do not prove, that the *C. raudensis* UWO241 IBPs are also derived from bacteria. The type II IBPs of *Chlamydomonas* CCMP681 may also have a bacterial origin. These proteins are characterized by numerous TXT motifs (where X is any amino acid) that have been implicated in ice-binding [4]. The closest matching sequence was from a bacterium from a hypersaline pond that also has numerous TXT motifs, which possibly serve to prevent water loss to the environment [4]. Together, these results provide further evidence that the IBP genes in polar algae have diverse origins unrelated to their taxonomic positions.

The large number of IBP isoforms in *C. raudensis* UWO241 are consistent with the findings in two other Antarctic ice-associated algae, the diatom *Fragilariopsis cylindrus* (T. Mock and J. Raymond, unpublished data) and the prymnesiophyte *Phaeocystis antarctica* (NCBI data submitted by P. Berg et al., Stanford University), both of whose genomes contain dozens of IBP and IBP-like genes. The high number of isoforms provides further evidence that the IBPs have an important role in survival under icy conditions.

The ice-binding proteins of *C. raudensis* are thus an additional tool in its repertory of adaptations for survival under harsh conditions of low light, high salinity and low temperature. We next plan to compare the transcriptomes of *C. raudensis* UWO241 and its mesophilic sister species SAG49.72 to better define the features that make UWO241 an Antarctic species.

## Supporting Information

Figure S1 ***E. coli*** **optimized codons for** ***Chlamydomonas raudensis*** **UWO 241 IBP isoform 1, without N-terminal signal peptide.**
(TIF)

Figure S2 **Analysis of recombinant** ***Chlamydomonas raudensis*** **IBP by GenScript (Piscataway, NJ).** A, SDS-PAGE gel. Lane 1, BSA; lane 2, recombinant protein. B, Western blot using anti-HIS antibody.
(TIF)

Figure S3 **Two-dimensional gel showing proteins purified from** ***C. raudensis*** **culture medium by ice-affinity.** Arrow indicates spot that matches pI and MW of sequenced ice-binding proteins. The spot above it may be a dimer. Y-axis, MW in kDa; x-axis, pI.
(TIF)

Figure S4 **Alignment of a putative ice-binding protein from** ***Stigmatella aurantiaca*** **and three isoforms of ice-binding proteins from** ***Chlamydomonas raudensis*** **UWO241.** Underlined sequences at N-terminus are predicted signal peptides. Isoform 3 also has a 117-amino acid sequence that goes between the DD residues marked by asterisks, which was removed to reduce the size of the figure.
(TIF)

Figure S5 **Agarose gel showing PCR products amplified from DNAs from the Antarctic** ***Chlamydomonas raudensis*** **UWO241 and the mesophilic** ***Chlamydomonas raudensis*** **SAG49.72, using primers for the following genes.** Lanes 1, IBP isoform 1; lanes 2–4, IBP isoform 2; lanes 6, RUBISCO (control); lanes 7, 18S rRNA (control). Right lane, DNA ladder.
(TIF)

Figure S6 **Neighbor-joining tree constructed from 18S rRNA sequences of ice-associated algae.** Tree is rooted on the 18S sequence of the rhodophyte *Cyanidioschyzon merolae*. Numbers at nodes indicate bootstrap values for 500 replications. Values less than 50 are not shown. Colors: Light green, diatoms; dark green, chlorophytes; brown, haptophyte; red, rhodophyte.
(TIF)
