# Supplementary figures and images for: Separate Origins of Ice-Binding Proteins in Antarctic Chlamydomonas Species
Source: PLoS One. 2013 Mar 11;8(3):e59186. doi: 10.1371/journal.pone.0059186 (PMC3594216; doi:10.1371/journal.pone.0059186)

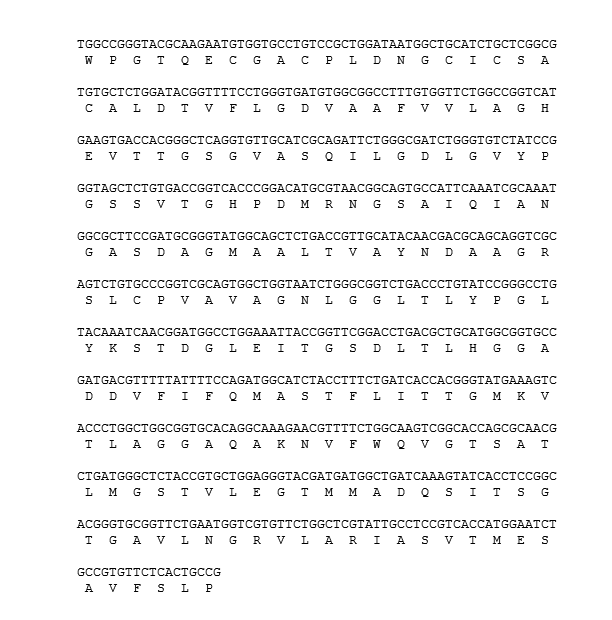

Supplement: Figure S1 — E. coli optimized codons for Chlamydomonas raudensis UWO 241 IBP isoform 1, without N-terminal signal peptide. (TIF) [file pone.0059186.s001.tif]

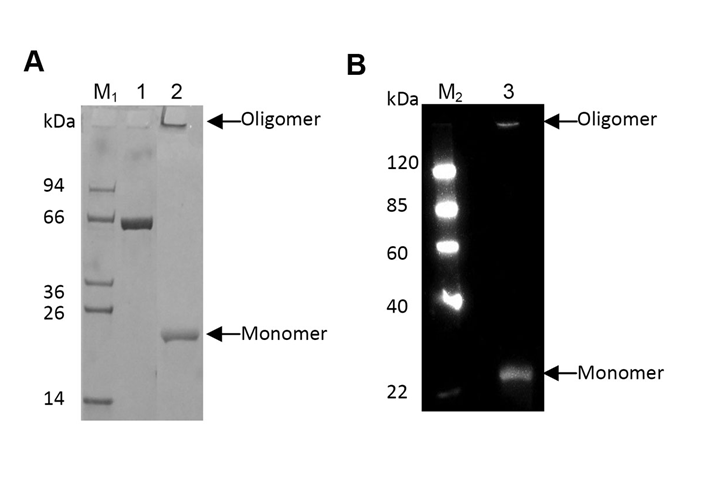

Supplement: Figure S2 — Analysis of recombinant Chlamydomonas raudensis IBP by GenScript (Piscataway, NJ). A, SDS-PAGE gel. Lane 1, BSA; lane 2, recombinant protein. B, Western blot using anti-HIS antibody. (TIF) [file pone.0059186.s002.tif]

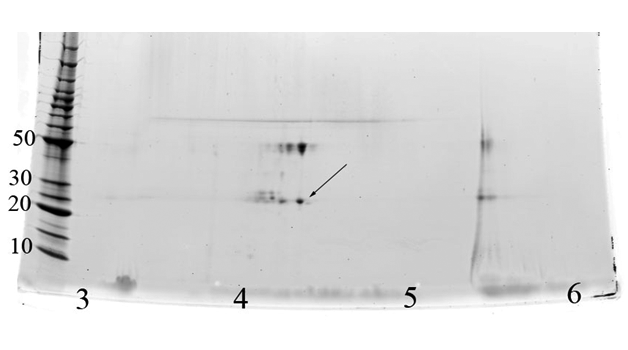

Supplement: Figure S3 — Two-dimensional gel showing proteins purified from C. raudensis culture medium by ice-affinity. Arrow indicates spot that matches pI and MW of sequenced ice-binding proteins. The spot above it may be a dimer. Y-axis, MW in kDa; x-axis, pI. (TIF) [file pone.0059186.s003.tif]

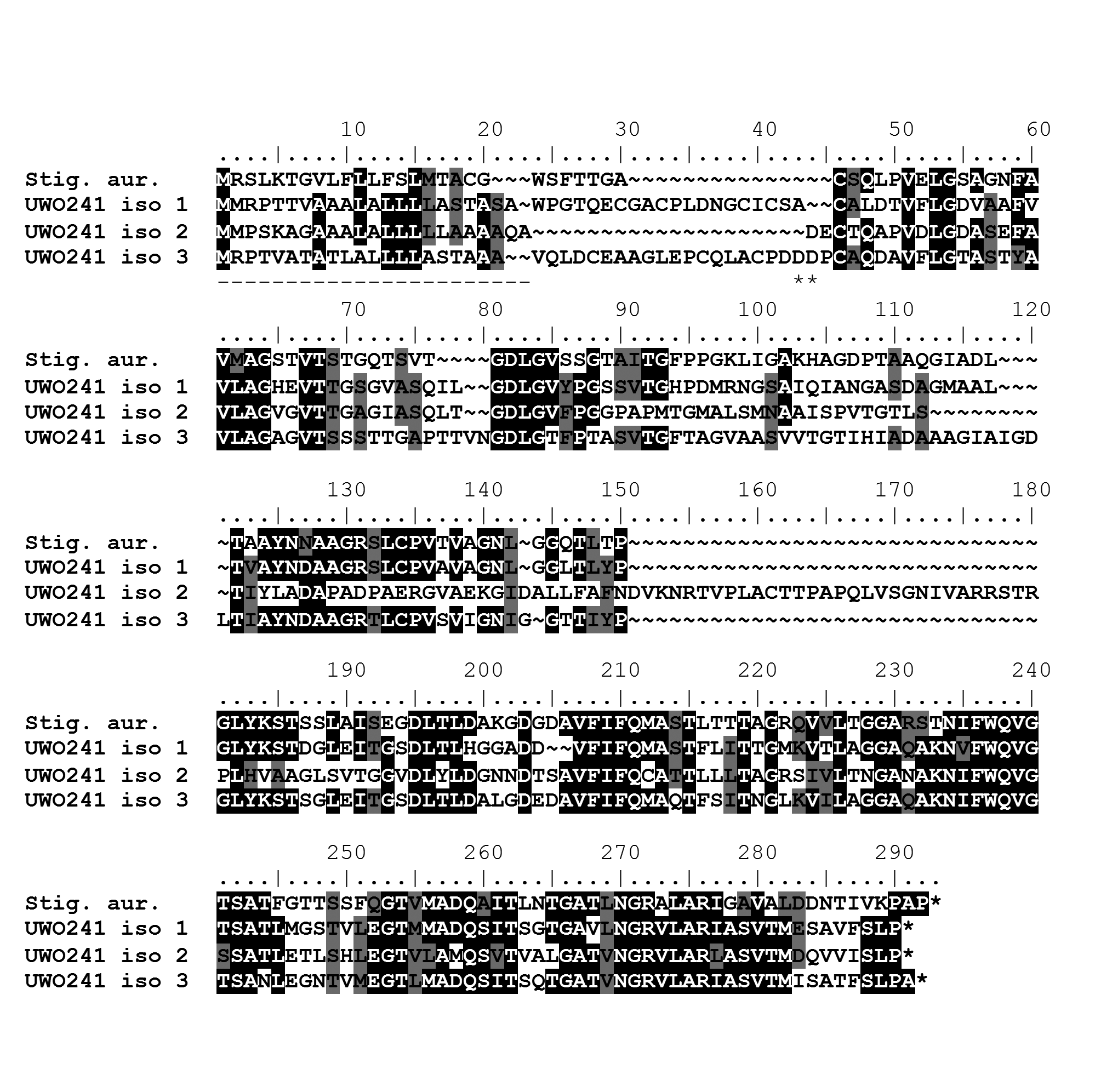

Supplement: Figure S4 — Alignment of a putative ice-binding protein from Stigmatella aurantiaca and three isoforms of ice-binding proteins from Chlamydomonas raudensis UWO241. Underlined sequences at N-terminus are predicted signal peptides. Isoform 3 also has a 117-amino acid sequence that goes between the DD residues marked by asterisks, which was removed to reduce the size of the figure. (TIF) [file pone.0059186.s004.tif]

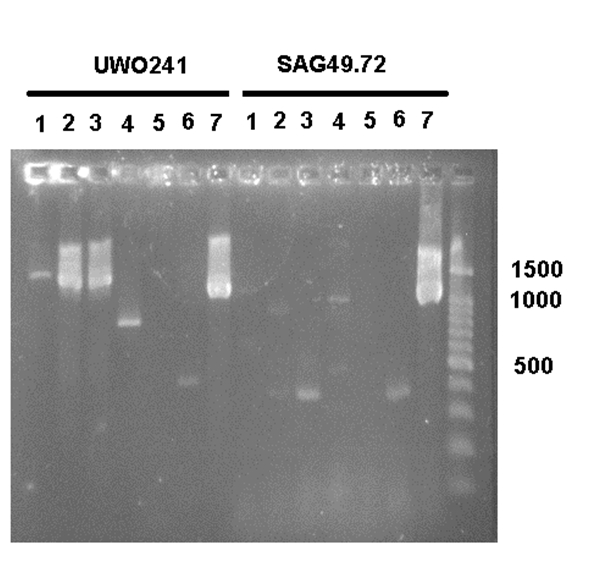

Supplement: Figure S5 — Agarose gel showing PCR products amplified from DNAs from the Antarctic Chlamydomonas raudensis UWO241 and the mesophilic Chlamydomonas raudensis SAG49.72, using primers for the following genes. Lanes 1, IBP isoform 1; lanes 2–4, IBP isoform 2; lanes 6, RUBISCO (control); lanes 7, 18S rRNA (control). Right lane, DNA ladder. (TIF) [file pone.0059186.s005.tif]

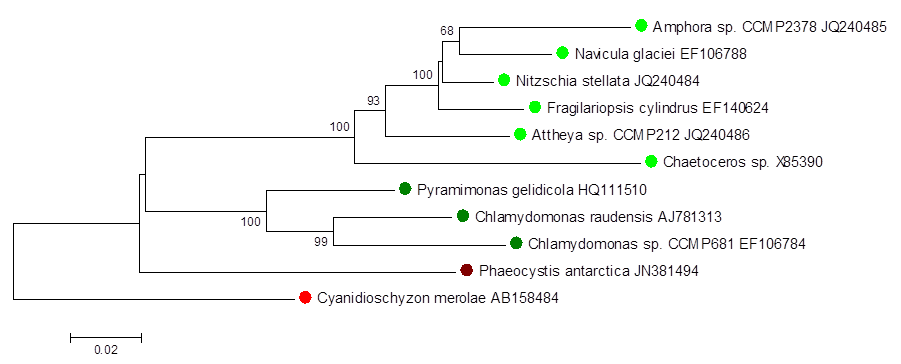

Supplement: Figure S6 — Neighbor-joining tree constructed from 18S rRNA sequences of ice-associated algae. Tree is rooted on the 18S sequence of the rhodophyte Cyanidioschyzon merolae. Numbers at nodes indicate bootstrap values for 500 replications. Values less than 50 are not shown. Colors: Light green, diatoms; dark green, chlorophytes; brown, haptophyte; red, rhodophyte. (TIF) [file pone.0059186.s006.tif]
